# Hereditary Angioedema With a Normal Complement Level

**DOI:** 10.7759/cureus.52291

**Published:** 2024-01-15

**Authors:** Nidal D Muna, Taimeh A Ahmed, Seham K Madaka, Tareq Z Nimer, Shatha I Hamdan, Sara N Ghaith, Tamara J Alshaer, Mohammad Naqib

**Affiliations:** 1 Faculty of Medicine, Al-Quds University, Abu Dis, PSE; 2 Paediatrics, Palestine Medical Complex, Ramallah, PSE

**Keywords:** case report, immunology, down syndrome, normal complement, hereditary angioedema

## Abstract

Hereditary angioedema (HAE) is an uncommon autosomal dominant disorder, characterized by episodes of oropharyngeal, gastrointestinal, and subcutaneous tissue swelling, often accompanied by discomfort. HAE is primarily associated with mutations in the SERPING1 gene, resulting in insufficient levels or impaired function of C1 esterase inhibitor (C1-INH), an important regulatory protein of the complement system. While types 1 and 2 HAE are well-established entities caused by quantitative and qualitative defects in C1-INH, respectively, the emergence of type 3 HAE, also known as estrogen-dependent HAE, has expanded our understanding of this complex disorder. In this case, a 2-year-old girl with Down syndrome visited the ER after experiencing lip and tongue swelling following the ingestion of ground pepper. Her laboratory results showed that her complement levels were within normal limits despite clinical symptoms. This situation leads to the specific variant of hereditary angioedema called hereditary angioedema with a normal C1 esterase inhibitor (HAE-NI-C1-INH). Although there are currently no approved treatments, positive responses have been seen to her use of C1-INH concentrate and tranexamic acid to alleviate both immediate and delayed symptoms.

## Introduction

Hereditary angioedema is an extremely serious and perhaps fatal medical disorder [1]. As an autosomal dominant condition, it causes frequent episodes of discomfort, swelling in the oropharynx, gastrointestinal tract, and subcutaneous tissue, and a lower quality of life [2]. 

The case at hand revolves around a Palestinian girl who presented with symptoms similar to hereditary angioedema. Of note, her laboratory results showed that her complement levels were within normal limits despite clinical symptoms. This situation leads to a specific variant of hereditary angioedema called hereditary angioedema with a normal C1 esterase inhibitor (HAE-NI-C1-INH). The diagnosis of this subtype includes the development of recurrent episodes of angioedema, maintenance of typical blood levels of C4 and C1-INH antigens, functional C1-INH activity, identification of genetic mutations, and consideration of family history [3].

Although there are currently no approved treatments, positive responses have been seen to her use of C1-INH concentrate and tranexamic acid to alleviate both immediate and delayed symptoms [4].

## Case presentation

A two-year-old Palestinian girl with Down syndrome presented to the emergency room (ER) on May 25, 2023, by her family, complaining of swelling in her lips and tongue after ingesting ground pepper. The patient had no known drug or food allergy and no previous history of similar episodes or past medical history apart from Down syndrome. A family history of similar episodes is unknown.

She was administered dexamethasone and chlorpheniramine maleate, which led to some improvement. As a result, she was discharged with an oral prescription of dimetindene.

The following day, the swelling increased along with mild tachypnea, prompting her family to bring her back to the ER. At the ER, she appeared conscious but irritable, with an O_2_ saturation of 84% and a blood pressure of 114/67. She was given chlorpheniramine maleate and received two doses of subcutaneous adrenaline and dexamethasone. Despite these efforts, there was no improvement. Hereditary angioedema was suspected, and subsequently, the patient was transferred to the pediatric intensive care unit (PICU) for further management. As the swelling continued to worsen, the decision was made to perform elective intubation in the operating room. She remained on synchronized intermittent mandatory ventilation (SIMV) for three days, and extubation was successfully performed on the fourth day.

**Figure 1:**
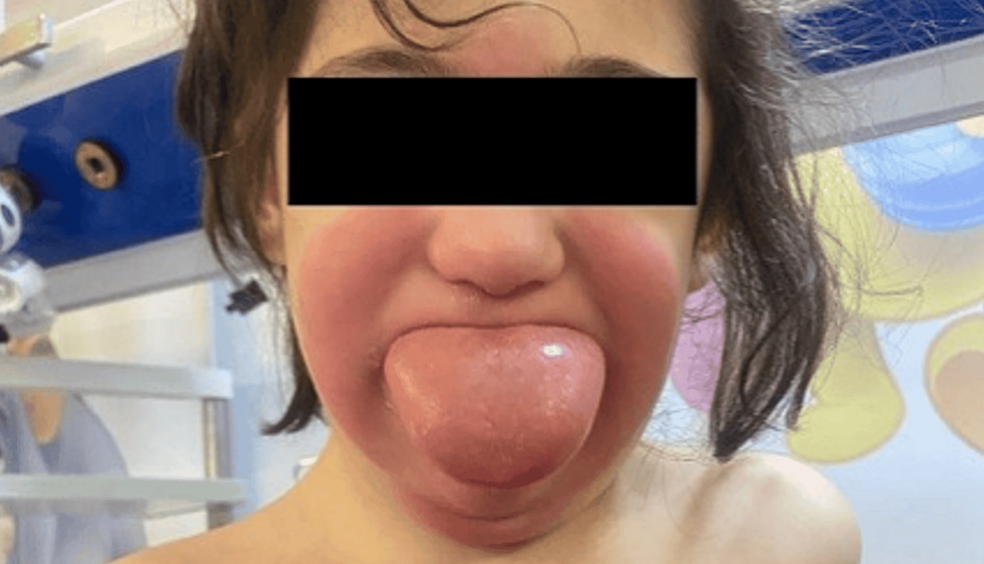
Facial, lip, and tongue swelling with perioral erythema

During the examination, her vital signs were as follows: temperature 36.4°C (97.52°F), heart rate 146 bpm, O_2_ saturation 98% on room air, and blood pressure 120/89. Her weight was 17 kg. Lung auscultation revealed clear bilateral air entry without any added sounds, heart auscultation identified normal heart sounds with no added sounds or murmurs. Her abdomen was soft and lax, with no palpable masses. The patient exhibited facial, lip, and tongue swelling, accompanied by perioral erythema (Figure 1). 

Upon admission to the PICU, a blood culture was obtained for sepsis workup, and intravenous ceftriaxone was initiated. On the fourth day, blood cultures exhibited growth of Streptococcus pyogenes. A subsequent control blood culture was conducted prior to initiating a new antibiotic regimen based on the latest results. An echocardiogram was performed, revealing trivial tricuspid regurgitation consistent with the ongoing infection.

Laboratory tests indicated a hemoglobin level of 7.7. Additionally, the C1 esterase level was 40 (normal range: 3-70), the C3 level was 159 (normal range: 88-155), and the C4 level was 42 (normal range: 12-32). Fresh frozen plasma (FFP) was administered, and she continued on a dose of 10 cc/kg every eight hours due to observed improvement. DNA samples were collected for genetic analysis prior to initiating packed red blood cells (PRBCs) transfusion. PRBCs were administered along with potassium replacement to address her hemoglobin levels.

A consultation with a pediatric immunologist was arranged. However, due to the necessity of a specific drug called "Cinryze," which was unavailable at the current hospital, the decision was made to transfer the patient to another medical facility.

## Discussion

HAE is primarily associated with mutations in the SERPING1 gene, resulting in insufficient levels or impaired function of C1 esterase inhibitor (C1-INH), an important regulatory protein of the complement system. While types 1 and 2 HAE are well-established entities caused by quantitative and qualitative defects in C1-INH, respectively, the emergence of type 3 HAE, also known as estrogen-dependent HAE, has expanded our understanding of this complex disorder [5].

Type 3 HAE presents unique challenges in diagnosis and management due to its distinct clinical and genetic characteristics. Unlike types 1 and 2 HAE, patients with type 3 HAE often have normal C1-INH levels and function. This poses a diagnostic dilemma, as clinicians typically rely on reduced C1-INH levels to confirm the diagnosis of HAE. However, it is crucial to consider type 3 HAE in cases where patients exhibit angioedema symptoms but have normal complement levels [5].

HAE attacks typically begin in children and worsen around puberty, but they can occur at any age. The emergence of symptoms in adulthood is substantially earlier in HAE type III individuals [6]. HAE Type 3 primarily affects women and is often associated with hormonal influences such as estrogen fluctuations during the menstrual cycle, pregnancy, and the use of hormonal contraceptives [7].

The most common symptom of hereditary angioedema type III is skin swelling. They appear most frequently on the face, much less frequently on the extremities, and just rarely on the genitals. Swellings of the extremities are more common in hereditary angioedema types I and II than swellings of other skin sites; facial and vaginal swellings occurred in only 3% and 4% of all skin swellings in hereditary angioedema caused by C1-INH deficiency, respectively [8]. Facial swellings occur at a far higher frequency than extremity swellings in practically all patients with hereditary angioedema type III, and this is an important and unequivocal diagnostic tip to this disorder. 

The initial presentation of the patient with swelling of the lips and tongue raised suspicion of an allergic reaction, especially given the history of ingestion of pepper. However, the persistence and worsening of symptoms despite initial treatment with dexamethasone and antihistamines necessitated a broader diagnostic approach. The presence of Down syndrome itself introduces complexities in symptom interpretation, as these individuals often have unique immunological and genetic features that can influence their response to various stimuli.

The identification of Streptococcus pyogenes growth in blood cultures underscores the potential overlap between infectious processes and angioedema. The sepsis component in this case further complicated the clinical picture, as sepsis-related inflammatory responses can contribute to angioedema development [9].

The refractory nature of the angioedema to initial treatments prompted an immunology consultation. The differentiation between allergic and non-allergic causes of angioedema is crucial, as it directs treatment strategies. The suspicion of hereditary angioedema (HAE) in this case emphasizes the need for an astute clinician to consider genetic factors. The measurement of C1 esterase levels and complement components C3 and C4 aids in the diagnosis of HAE.

HAE treatment comprises both acute attack management and long-term prophylaxis. It has been proven that corticosteroids, antihistamines, and epinephrine are ineffective [7]. These medications can be utilized in the acute phase: concentrated INCH1, fresh frozen plasma, ecallantide, and icatibant [7]. Prophylactic therapy consists of using attenuated androgens, antifibrinolytic medicines, and progesterone. Because they enhance the levels of INHC1 and minimize the frequency of crises, attenuated androgens are the most effective and well-tolerated medications [10].

Administration of FFP to the child led to a significant improvement in facial swelling, pointing to the potential benefit of plasma-derived factors in ameliorating angioedema symptoms. The decision to use FFP as a therapeutic option reflects the integration of both clinical judgment and available evidence in managing a complex case like this. Further research is warranted to elucidate the mechanisms underlying FFP's effectiveness and to determine its optimal role in the treatment algorithm for angioedema.

The case presents an opportunity to explore potential genetic factors contributing to the patient's condition. Saving a DNA sample for possible genetic workup reflects the forward-thinking approach to understanding the underlying mechanisms of angioedema in this context [11].

## Conclusions

This case illustrates hereditary angioedema presenting with a normal complement level, especially C1-INH levels and function. Physicians often use the complement level to diagnose HAE, causing a diagnostic dilemma for cases like ours. The treatment approach involves both acute attack management and long-term prophylaxis. Concentrated INCH1, fresh frozen plasma, ecallantide, and icatibant often constitute acute attack management. Prophylactic therapy often consists of attenuated androgens, antifibrinolytics, and progesterone.
